# Investigation of the Effect of TiO_2_ as a Dietary Marker on Broiler Intestinal Fermentation: Combination of Ex Vivo Simulation and In Vivo Approach

**DOI:** 10.3390/ani16121867

**Published:** 2026-06-17

**Authors:** Ali Kiani, German Jurgens, Gemma Gonzalez-Ortiz, Carrie L. Walk, Teemu Rinttilä

**Affiliations:** 1 Alimetrics Research Oy, FI-02920 Espoo, Finland; 2 AB Vista, Marlborough SN8 4AN, UK

**Keywords:** titanium dioxide, gut microbiota, intestinal fermentation, ex vivo model

## Abstract

The impact of dietary inert digestibility markers on gut microbiota and intestinal fermentation remains poorly understood. This study investigated the effects of dietary titanium dioxide (TiO_2_) supplementation at 4 kg/t feed, representing a typical dose used in animal nutrition studies, on fermentation dynamics and microbial composition in broiler chickens using combined ex vivo and in vivo approaches. Ex vivo fermentations were conducted using ileal and caecal microbiota and substrates collected from 32-day-old broiler chickens. Titanium dioxide (TiO_2_) was supplemented directly to the fermentations, and gas production and short-chain fatty acid (SCFA) profiles were used as the main outcome measures. In parallel, 392 broiler chickens were fed diets with or without TiO_2_ for 32 days, and ileal and caecal digesta were analysed for fermentation end-products and microbial composition using shotgun metagenomic sequencing. A second ex vivo experiment was performed using microbiota adapted to dietary TiO_2_. In the first ex vivo model, TiO_2_ reduced gas production and acetic acid concentration in the ileum (*p* < 0.05), whereas in the caecum it increased gas production, total eubacterial counts, and branched-chain fatty acids (BCFAs) (*p* < 0.05). In vivo, TiO_2_ did not affect growth performance or organ development but significantly increased isobutyric acid and total BCFA concentrations in the caecum (*p* < 0.05). Metagenomic analysis revealed increased caecal alpha diversity (Shannon index) and enrichment of taxa associated with amino acid metabolism, including *Massilicoli timonensis*, *Blautia merdavium*, *Rubneribacter badeniensis*, and *Mediterraneibacter caccavium*. The second ex vivo experiment showed similar trends, with increased gas and BCFA production. Collectively, these findings indicate that TiO_2_ can modulate intestinal fermentation and microbial composition in a segment-specific manner, suggesting that dietary markers may not be biologically inert.

## Simple Summary

Inert substances used to measure digestion in animal studies are generally assumed to be inactive, but their effects on intestinal microbiota and metabolism are not fully understood. This study examined whether titanium dioxide, a commonly used feed marker, influences gut bacteria in broiler chickens. Using both laboratory and animal experiments, titanium dioxide was found to reduce fermentation in the ileum but increase it in the caecum. It did not affect growth performance or overall health. However, it altered fermentation patterns and increased microbial diversity in the lower gut, including bacteria associated with protein breakdown. Overall, these findings suggest that titanium dioxide can influence gut microbial activity, indicating that such markers may not be completely inert and should be used with caution.

## 1. Introduction

Inert digestibility markers, such as titanium dioxide (TiO_2_), are widely used in animal nutrition research to estimate nutrient utilisation and digestibility along the gastrointestinal tract. Dietary markers offer a practical alternative to labour-intensive total collection methods, streamlining digestibility studies. However, concerns remain regarding variability in marker recovery rates, potential interactions with digestive enzymes [1,2], altered intestinal barrier function [3], and species-specific responses to dietary formulations [4]. Additionally, recent findings suggest that gut microbiota and metabolic pathways may be influenced by dietary markers [5], highlighting the need for deeper investigation into their biological effects.

Emerging evidence indicates that TiO_2_ nanoparticles can disrupt gut microbiota composition and functionality. Oral exposure to TiO_2_ has been shown to alter microbial community structure, including reductions in genera commonly associated with beneficial metabolic functions, such as *Prevotella* and *Bacteroides* [6,7,8]. Furthermore, TiO_2_ exposure has been linked to increased intestinal permeability, microbial translocation, and immune activation [9,10], as well as oxidative stress responses in gut bacteria [5,11]. Animal studies have demonstrated that food-grade TiO_2_, even at physiological doses, can profoundly affect gut homeostasis, microbial composition, and fermentation processes in the small intestine [12]. These findings suggest that dietary markers may influence intestinal fermentation, particularly the production of short-chain fatty acids (SCFAs), which are vital for gut health and energy metabolism.

Studying the mechanisms and effects of dietary markers in animals is challenging due to multiple interacting processes. In well-functioning biological systems, intermediate metabolites rarely accumulate because metabolic pathways are tightly coupled, with production and conversion rates closely matched. This makes it difficult to assess individual reaction kinetics. In vivo studies are further limited by inter-individual variation in gut microbiota and the rapid absorption of transient metabolites, which complicates monitoring of fermentation kinetics. Given these limitations, ex vivo fermentation systems offer a robust and ethical alternative for investigating microbial activity and community-level adaptations. These systems allow for the transplantation of intact microbial communities into anaerobic culture vessels, enabling precise control of environmental conditions and real-time monitoring of fermentation dynamics without confounding epithelial absorption effects [13,14]. Therefore, improving existing ex vivo models to better replicate physiological conditions enhances their relevance for studying microbial responses to dietary compounds.

It is notable that no previous studies have specifically examined the impact of dietary TiO_2_ on the intestinal microbiota and SCFA profiles of broilers. To investigate the impact of TiO_2_ on fermentation and gut microbiota in broilers, a combination of ex vivo and in vivo approaches were employed to determine whether dietary markers influence the composition and fermentation dynamics of broiler gut microbiota. Shotgun metagenomic sequencing was employed to evaluate microbiota composition, providing insights into how TiO_2_ influence fermentation dynamics and microbiota composition along the intestinal tract.

## 2. Materials and Methods

### 2.1. Ex Vivo Experiment I

#### 2.1.1. Broiler Ileal and Caecal Ex Vivo Fermentation Models

Ileal and caecal ex vivo fermentation experiments were conducted to mimic the luminal environment of the ileum and caecum in broiler chickens. To create an authentic substrate medium for the ileal fermentation model, intestinal contents were collected from the distal jejunum to Meckel’s diverticulum of 30 Ross 308 broiler chickens at 32 d of age. All birds were fed wheat–soybean-based diets formulated to meet the nutrient requirements of Ross 308 broilers, identical to the control diet (described in Supplementary Table S1). The collected intestinal contents were pooled and thoroughly homogenised to ensure uniformity of the substrate medium for subsequent ex vivo fermentation experiments.

For the caecal fermentation, the substrate was prepared by combining distal ileal and caecal contents of the same birds. The ileum was defined as the intestinal segment extending from Meckel’s diverticulum to approximately 2 cm proximal to the ileocaecal junction, and digesta from both caecal lobes were pooled for sample preparation.

The jejunal digesta preparation (ileal model) and the combined ileal/caecal digesta preparation (caecal model) were mixed with an equal volume of anaerobic buffer (pH 6.85; 0.02 M K_2_HPO_4_, 0.02 M NH_4_H_2_PO_4_, and 0.6 mmol MgSO_4_) and centrifuged at 18,000× *g* for 20 min to remove solid digesta particles and most bacterial cells. The pellet was discarded, and the clarified supernatants were used as the bacterial growth substrates in the ex vivo experiment. The microbial inocula used in the ileal and caecal fermentation models consisted of fresh ileal or caecal digesta collected from 35-d-old Ross 308 broilers fed the same wheat–soybean-based diet without additives. No antibiotics, coccidiostats or feed additives were included in these diets, except for 500 FTU/kg of exogenous phytase. For inocula preparation, 20 control diet-fed birds were sacrificed, and ileal and caecal contents were collected and maintained under anoxic conditions to retain viability of the bacteria until they were used for the inoculation of the simulation vessels (within 2 h of sacrificing the birds).

The ileal simulation was initiated by adding 5.0 mL of the previously described substrate medium, either 30 mg of TiO_2_ (TiO_2_ treatment) or no TiO_2_ (control treatment), 0.1 g of ileal digesta inoculum of individual birds, and 5.0 mL of pH 6.85 buffer solution (20 mM K_2_HPO_4_, 20 mM NH_4_H_2_PO_4_, 0.6 mM MgSO_4_) to 20-mL serum bottles in an anaerobic chamber.

The caecal simulation was initiated by adding 5.0 mL of the substrate medium as previously described, either 30 mg of TiO_2_ (TiO_2_ treatment) or no TiO_2_ (control treatment), 0.5 g of caecal digesta inoculum of individual birds and 5.0 mL of pH 6.85 buffer solution (20 mM K_2_HPO_4_, 20 mM NH_4_H_2_PO_4_, 0.6 mM MgSO_4_) to 20-mL serum bottles in an anaerobic chamber. To replicate genuine caecum conditions, the buffer was reduced to −340 mV with a mixture of NaS and cysteine.

Simulation vessels were sealed with thick butyl rubber stoppers, maintained at 42 °C, and continuously mixed on a gyratory shaker at 100 rpm. Each treatment was replicated in five vessels. Inoculations were performed in a random order to minimise potential systematic bias associated with inoculation timing or inoculum freshness. Ileal and caecal simulation vessels were incubated for 8 h and 9 h, respectively, after which samples were collected for subsequent analyses as described below.

#### 2.1.2. Gas Production Measurement

Gas production was measured in the simulation vessels at 5-h and 8-h time points for ileal model and 5-h and 9-h time points for caecal model by manually puncturing the rubber stopper with a needle that was connected to a high-precision glass syringe with a sensitive ground plunger and recording the volume of total gas released from the fermentation vessels.

#### 2.1.3. Fermentation End-Product Analysis

Volatile fatty acids (VFAs) and lactic acid were quantified as free acids in ileal simulation samples after 8 h and caecal simulation samples after 9 h of incubation using a previously described gas chromatography method [15]. Briefly, samples were prepared using pivalic acid as an internal standard and analysed by gas chromatography with flame ionisation detection (Agilent Technologies, Santa Clara, CA, USA). The quantified acids included acetic, propionic, butyric, valeric, isobutyric, 2-methylbutyric, isovaleric, and lactic acid. Concentrations below the laboratory detection limit were considered non-detectable.

#### 2.1.4. DNA Extraction and Total Eubacterial Quantification by qPCR

Subsamples from the ileal and caecal ex vivo experiments were collected for quantification of total eubacterial load by real-time quantitative PCR (qPCR) as previously described [16]. Briefly, microbial DNA was extracted from 0.4 mL of simulation sample using bead-beating and phenol–chloroform extraction, followed by isopropanol precipitation and resuspension in Tris-EDTA buffer.

Total eubacterial load was quantified using a 16S rRNA gene-targeted qPCR assay. Amplifications were performed using a QuantStudio™ 7 Flex Real-Time PCR System (Applied Biosystems, Foster City, CA, USA) in 15 μL reaction volumes containing SYBR™ Select Master Mix (Applied Biosystems), 0.25 μM of each forward and reverse primer, and 5 μL of 1:1000-diluted template DNA. Deionised sterile water served as a no-template control. The primer sequences (5′–3′) were F: TCCTACGGGAGGCAGCAGT and R: GGACTACCAGGGTATCTAATCCTGTT [17], generating an amplicon of approximately 465 bp. Quantification was performed using a standard curve generated from ten-fold serial dilutions of synthetic 16S rRNA gene fragments (gBlocks^®^ Gene Fragments, IDT, Coralville, IA, USA). Results were normalised to the amount of starting material and log10-transformed prior to statistical analysis.

### 2.2. In Vivo Study

#### 2.2.1. Animal Ethics Statement

All experimental procedures were conducted in accordance with the European Union guidelines for the care and use of animals in research and Directive 2010/63/EU on the protection of animals used for scientific purposes [18]. The feeding trial with broiler chickens was carried out at the licensed research facility of Alimetrics Research Ltd. located in Koski Tl, Finland. The same experimental diets used in ex vivo experiment 1 were also used in the in vivo study. The diets were nutritionally similar across treatments (Supplementary Table S1), except for TiO_2_ added at 4 g/kg.

#### 2.2.2. Husbandry Practices

A total of 392 day-old male Ross 308 broiler chicks were weighed and randomly allocated to two treatment groups: T1 (without TiO_2_) and T2 (with TiO_2_), with 196 chicks in each group. Each treatment group consisted of 14 replicate pens, with 14 birds placed in each 1.12 m^2^ floor pen on clean wood shavings. The maximum stocking density of 33 kg/m^2^ specified by welfare law was not exceeded over the rearing period. Birds were allowed ad libitum access to the treatment diets and water for the duration of the trial. The room was thermostatically controlled to produce an initial temperature of 32 °C on day 1, which was reduced during the study to 22 °C by day 21. The lighting regimen consisted of 24 h of light on day 1, followed by a gradual increase in darkness over the subsequent week until 6 h of darkness was reached, which was maintained for the remainder of the study.

#### 2.2.3. Data and Sample Collection

Initial pen body weight (BW) was recorded at placement. Thereafter, feed intake and BW were recorded by pen at 14, 28, and 32 d of age. Birds were observed twice daily, and mortalities were removed and weighed to calculate feed conversion ratio (FCR) based on total BW produced.

On days 14, 21, and 32 of the study, two birds per pen were randomly selected for internal organ sampling. Prior to euthanasia by cervical dislocation, birds were individually tagged, and live body weight was recorded. Post-mortem, the crop, gizzard, and liver were excised and weighed. The crop and gizzard were emptied and cleaned prior to weighing. In addition, the small intestine was removed, emptied of its contents, and measured for length and weight. Absolute organ weights (g) were recorded, and relative organ weights (g/kg BW) were subsequently calculated by dividing each organ weight by BW. Absolute intestinal lengths (cm) of the duodenum, jejunum, and ileum were recorded, and relative intestinal length was expressed as the ratio of intestinal segment length to BW (cm/kg BW).

#### 2.2.4. Metagenomic Sequencing and Fermentation End-Products in Ileal and Caecal Digesta

Sampling for metagenomic sequencing and fermentation end-product analysis of ileal and caecal digesta was conducted on day 32. A total of 24 birds were randomly selected from each treatment group. Each digesta sample was divided into two equal subsamples, one used for shotgun metagenomic sequencing and the other for analysis of VFA and lactic acid concentrations. DNA was extracted from the digesta samples using the method described in Section 2.1.4 (DNA Extraction and Total Eubacterial Quantification by qPCR). Shotgun metagenomic sequencing was performed by CMBIO (Germantown, MD, USA). VFA and lactic acid concentrations in ileal and caecal digesta were analysed as described in Section 2.1.3 (Fermentation End-Product Analysis).

#### 2.2.5. Microbiome Data Processing and Analysis

DNA libraries were prepared using the Watchmaker DNA Library Prep Kit (7K0019-1K; Watchmaker Genomics, Boulder, CO, USA) according to the manufacturer’s instructions. Genomic DNA was quantified using a Qubit™ dsDNA HS Assay Kit and a Qubit™ fluorometer (Thermo Fisher Scientific, Waltham, MA, USA). DNA was fragmented using Watchmaker Frag/AT buffer and enzyme mix, followed by adapter ligation using xGen™ Unique Dual Index (UDI) primers (Integrated DNA Technologies, Coralville, IA, USA) and Stubby adapters (Element Biosciences, San Diego, CA, USA). Libraries were amplified using seven PCR cycles, purified using CleanNGS magnetic beads (CleanNA B.V., Waddinxveen, The Netherlands), and quantified prior to sequencing. Circularisation was performed using the Adept Library Compatibility Kit (Element Biosciences, San Diego, CA, USA), and paired-end shotgun sequencing (2 × 150 bp) was performed on an AVITI™ sequencing system (Element Biosciences, San Diego, CA, USA), generating an average sequencing depth of approximately 20 million paired-end reads per sample.

Raw sequencing data were processed using the CosmosID-HUB cloud-based metagenomics platform (CosmosID Inc., Germantown, MD, USA; September 2025 pipeline release). Quality control included removal of low-quality reads, adapter trimming, and filtering of short and low-complexity reads using the default quality control parameters of the CosmosID-HUB pipeline. Host-derived reads (*Gallus gallus*) were removed through alignment to the host reference genome within the CosmosID pipeline prior to downstream analysis.

Taxonomic profiling was performed using the CosmosID Kepler k-mer–based classifier and the curated GenBook™ reference database integrated within the CosmosID-HUB platform (September 2025 release). Relative abundance profiles were generated using the built-in probabilistic abundance estimation workflow implemented in CosmosID-HUB.

Downstream analyses were performed using relative abundance data. Alpha diversity differences between control and TiO_2_-supplemented groups were assessed using Wilcoxon rank-sum tests. Beta diversity was evaluated using principal coordinates analysis (PCoA) based on Bray–Curtis dissimilarity, and group differences were tested using permutational multivariate analysis of variance (PERMANOVA). Reference-based comparative profiling was performed against a curated database of over 40,000 microbiome samples integrated within the platform.

Differentially abundant bacterial taxa were identified using Linear Discriminant Analysis Effect Size (LEfSe) implemented in CosmosID-HUB. Taxa with a Kruskal–Wallis test *p*-value < 0.038 and an LDA score > 3.23 were considered discriminative biomarkers. Unless otherwise specified, default CosmosID-HUB parameters were used throughout the analyses.

### 2.3. Ex Vivo Experiment II

The aim of the second ex vivo experiment was to evaluate the caecal fermentation model using substrate and inoculum obtained from birds with microbiota adapted to TiO_2_ feeding. Substrate and inoculum were collected from birds in the in vivo study at two distinct time points: on day 32, twenty birds from each treatment group were sacrificed to collect ileal and caecal digesta, and on day 35, an additional ten birds per treatment were sacrificed to collect the fresh caecal digesta for inoculum preparation. Each treatment was replicated in ten independent fermentation vessels.

The primary methodological distinction of the second ex vivo model was the source of the substrate and inoculum used in the fermentations. Vessels were established using substrate and inoculum from birds fed a diet without TiO_2_ (control treatment) or from birds fed a diet supplemented with TiO_2_ at 4 g/kg. All other procedures were performed identically to those described for ex vivo experiment I (Section 2.1).

### 2.4. Statistical Analysis

Statistical analyses were performed using JMP Pro 17 (SAS Institute Inc., Cary, NC, USA). Differences between the control and TiO_2_ treatments were assessed using two-tailed Student’s *t*-tests based on mean values. Assumptions of normality were evaluated by inspection of residual distribution plots. Microbiome sequencing data were analysed using the bioinformatic and statistical methods described in Section 2.2.5.

## 3. Results

### 3.1. Microbial and Fermentation Responses in Ex Vivo Experiment I

#### 3.1.1. Ileal Ex Vivo Fermentation Model

**Figure 1:**
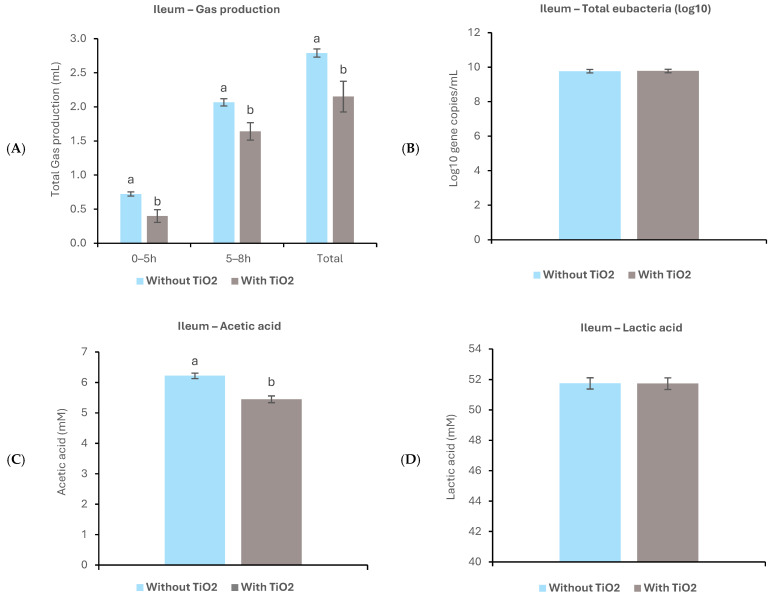
Effects of TiO_2_ supplementation on fermentation activity in ileal ex vivo model I. (**A**) Gas production (mL), (**B**) total eubacteria (log_10_ gene copies/mL), (**C**) acetic acid (mM), and (**D**) lactic acid (mM) measured at the end of the 8-h fermentation period (*n* = 5 per treatment). For gas production, columns show gas production during individual fermentation intervals and total cumulative gas production after 8 h of incubation. Significant differences (*p* < 0.05) are indicated by different letters above each column. Bars represent the standard error of the mean.

In the ileal ex vivo simulation, gas production was monitored over two intervals (0–5 h and 5–8 h) and cumulatively over the 0–8 h period (Figure 1A). Gas production was significantly lower in the TiO_2_ treatment compared with the control during both the initial 0–5 h and subsequent 5–8 h intervals (*p* < 0.05). Consequently, cumulative gas production over the full 0–8 h period was also reduced in the TiO_2_ treatment relative to the control (*p* < 0.05). These findings indicate that inclusion of TiO_2_ reduced overall fermentation activity in the ileal simulation model.

At the end of the 8-h ileal ex vivo fermentation period, no significant differences in total eubacterial concentrations were observed between the control and TiO_2_ treatments (Figure 1B). In contrast, TiO_2_ supplementation reduced acetic acid concentration compared with the control (*p* < 0.05; Figure 1C), whereas lactic acid concentration was not affected (Figure 1D).

#### 3.1.2. Caecal Ex Vivo Fermentation Model

**Figure 2:**
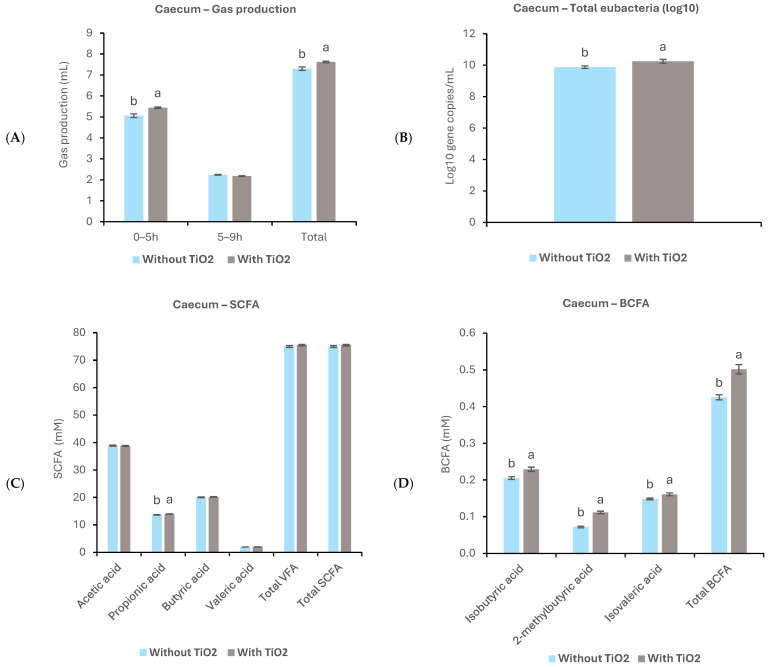
Effects of TiO_2_ on fermentation activity in caecal ex vivo model I. (**A**) Gas production (mL), (**B**) total eubacteria (log_10_ gene copies/mL), (**C**) acetic acid (mM), propionic acid (mM), butyric acid (mM), valeric acid (mM), total VFAs (mM), and total SCFAs (mM), and (**D**) isobutyric acid (mM), 2-methylbutyric acid (mM), isovaleric acid (mM) and total BCFAs (mM) measured at the end of the 9-h fermentation period (*n* = 5 per treatment). For gas production, columns show gas production during individual fermentation intervals and total cumulative gas production after 9 h of incubation. Significant differences (*p* < 0.05) are indicated by different letters above each column. Bars represent the standard error of the mean.

In the caecal ex vivo simulation, gas production was measured over the 9-h fermentation period (Figure 2A). Gas production was significantly higher in the TiO_2_ treatment compared to the control during the initial 0–5 h of incubation (*p* < 0.05). During the subsequent 5–9 h interval, gas production declined in both treatment groups, and no significant differences were observed between the treatment groups. Over the 0–9 h period, the cumulative gas production was significantly higher in the TiO_2_ treatment than the control (*p* < 0.05). These results indicate that TiO_2_ influenced the caecal fermentation dynamics, particularly during the early phase of incubation.

At the end of the 9-h caecal ex vivo fermentation period, total eubacterial counts were significantly higher in the TiO_2_ treatment than in the control (*p* < 0.05; Figure 2B). TiO_2_ supplementation also altered the SCFA profile in the caecal simulation (Figure 2C). Specifically, propionic acid concentration was significantly higher in the TiO_2_ treatment compared with the control (*p* < 0.05). Valeric acid, butyric acid, total VFA, and total SCFA concentrations were numerically higher, but did not differ significantly between the treatments. Moreover, TiO_2_ supplementation significantly increased the total branched-chain fatty acids (BCFAs) concentration compared with the control (*p* < 0.05). Concentrations of individual BCFAs, including isobutyric acid, 2-methylbutyric acid, and isovaleric acid, were also significantly higher in the TiO_2_ treatment (*p* < 0.05; Figure 2D).

### 3.2. In Vivo Performance and Physiological Responses

#### 3.2.1. Growth Performance

The in vivo study evaluated the impact of dietary TiO_2_ inclusion on feed intake (FI), body weight gain (BWG), and FCR in broilers across three growth phases: 0–14, 14–21, and 21–32 d of age. Growth performance was also evaluated for the 0–21 d and 0–32 d periods (Table 1 and Table 2).

No significant differences were observed between the dietary treatments for FI, BWG, mortality corrected FCR (mFCR), or BW from hatch to day 14 or during the 14–21 d period. However, birds fed a diet without TiO_2_ exhibited numerically lower mFCR compared to those receiving TiO_2_ supplementation, with this difference approaching statistical significance during the 0–14 d period (*p *= 0.061; Table 1).

A similar trend was observed during the 21–32 d period and across the entire experimental period (0–32 d) where birds fed diets without TiO_2_ consistently showed numerically lower mFCR compared with those fed TiO_2_-supplemented diets. This tendency also approached statistical significance during the 21–32 d interval (*p *= 0.099; Table 2).

Across the entire 0–32 d period, no differences in body weight gain were observed. However, during 21–32 d, gain tended to be greater in birds fed diets without TiO_2_ (*p* = 0.060). Final body weight followed a similar pattern with no significant effects from TiO_2_ (*p *= 0.610).

#### 3.2.2. Internal Organs

There were no statistically significant differences in the absolute or relative weight of the crop or relative jejunal length at different ages. At day 32, there was a tendency towards a significant difference (*p *= 0.051) in the relative length of the ileum, with birds fed diets containing TiO_2_ showing higher values compared with those fed diets without TiO_2_ (Table 3).

#### 3.2.3. VFA and Lactic Acid Concentrations in Ileal and Caecal Digesta

**Figure 3:**
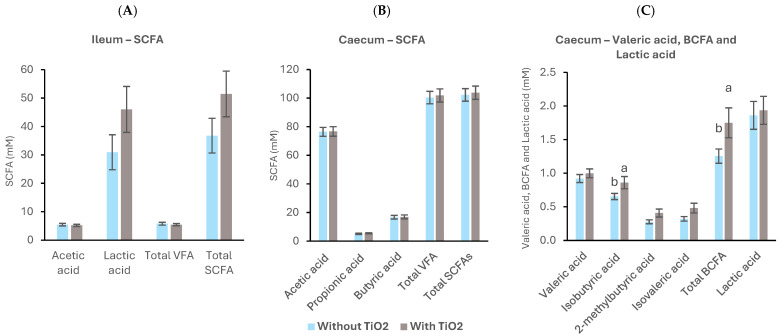
Effects of dietary TiO_2_ supplementation on VFAs and lactic acid concentration in ileal and caecal digesta on day 32. (**A**) Acetic acid (mM), lactic acid (mM), total VFA (mM) and total SCFA (mM) concentration in ileal digesta. (**B**) Acetic acid (mM), propionic acid (mM), butyric acid (mM), total VFA (mM), and total SCFA (mM) concentration in caecal digesta, and (**C**) valeric acid (mM), isobutyric acid (mM), 2-methylbutyric acid (mM), isovaleric acid (mM), total BCFA (mM) and lactic acid (mM) concentration in caecal digesta. Data are means of 14 replicate pens per treatment. Significant differences (*p* < 0.05) are shown as different letters above each column. Bars represent standard error of the mean.

In ileal digesta samples collected on day 32, TiO_2_ supplementation did not affect total SCFA or lactic acid concentrations (Figure 3A). In caecal digesta, propionic acid, butyric acid, total VFAs, and total SCFAs were likewise unaffected by TiO_2_ treatment compared with the control diet (Figure 3B). However, isobutyric acid and total BCFA concentrations were higher in birds fed the TiO_2_-supplemented diet than in birds fed the control diet (*p* < 0.05). Valeric acid, 2-methylbutyric acid, isovaleric acid, and lactic acid were not influenced by treatment (Figure 3C).

#### 3.2.4. Sequence Analysis

**Figure 4:**
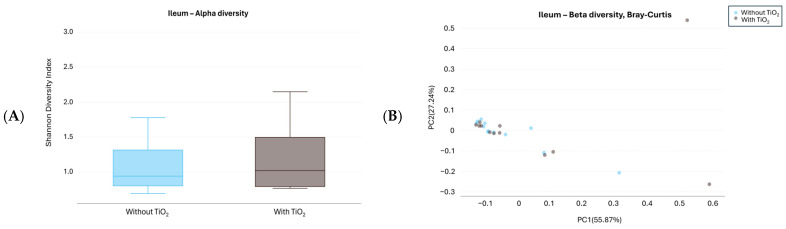
Microbial diversity in ileal digesta samples collected on day 32 from broilers fed diets with or without TiO_2_ supplementation. (**A**) Box plot of alpha diversity (Shannon index), which accounts for both species’ richness (the total number of different species present) and evenness (the relative abundance of different species) within a sample. (**B**) Beta diversity PCoA plot based on Bray-Curtis distances, illustrating differences in microbial community composition between samples from birds fed diets with and without TiO_2_ supplementation based on species relative abundances.

**Figure 5:**
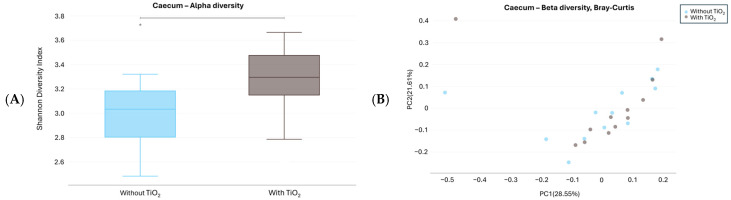
Microbial diversity in caecal digesta samples collected on day 32 from broilers fed diets with or without TiO_2_ supplementation. (**A**) Box plot of alpha diversity (Shannon index), which accounts for both species’ richness (the total number of different species present) and evenness (the relative abundance of different species) within a sample. An asterisk indicates significant difference between the treatments (*p* < 0.05; *n* = 14 per treatment). (**B**) Beta diversity PCoA plot based on Bray-Curtis distances, illustrating differences in microbial community composition between samples from birds fed diets with and without TiO_2_ supplementation based on species relative abundances.

In ileal digesta samples collected on day 32, no significant differences in alpha diversity were observed, as assessed by the Shannon diversity index (Figure 4A). In caecal digesta samples collected on day 32, metagenomic sequencing revealed a significant increase in alpha diversity in birds fed the TiO_2_-supplemented diet, as indicated by the Shannon diversity index (*p* = 0.021; Figure 5A). In contrast, the Chao1 index showed a numerical increase that approached statistical significance (*p* = 0.057; Supplementary Figure S1A), whereas the Simpson index indicated no significant differences between treatments (Supplementary Figure S1B).

Species-level beta-diversity analysis based on Bray–Curtis distances indicated no significant differences in microbial community composition between treatments in either ileal (Figure 4B) or caecal (Figure 5B) digesta samples, as assessed by PERMANOVA.

**Figure 6:**
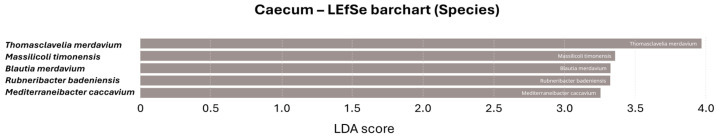
Bacterial species significantly enriched in caecal digesta samples collected on day 32 from broilers fed the TiO_2_-supplemented diet, as identified by Linear Discriminant Analysis Effect Size (LEfSe) analysis (LDA score > 3.23; *p* < 0.038).

**Figure 7:**
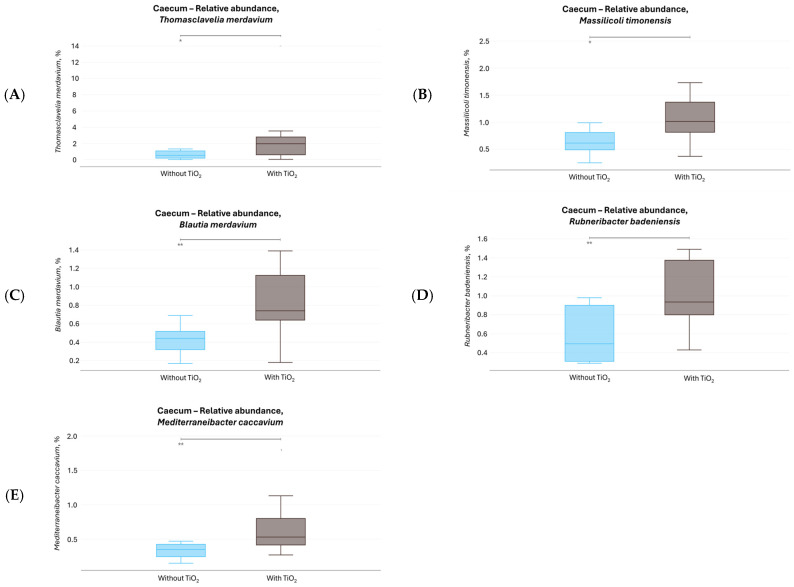
Relative abundances of five bacterial species identified by LEfSe as significantly enriched in caecal digesta samples collected on day 32 from broilers fed the TiO_2_-supplemented diet: (**A**) *Thomasclavelia merdavium* (*p* = 0.024), (**B**) *Massilicoli timonensis* (*p* = 0.028), (**C**) *Blautia merdavium* (*p* = 0.006), (**D**) *Rubneribacter badeniensis* (*p* = 0.008), and (**E**) *Mediterraneibacter caccavium* (*p* = 0.003). Asterisks indicate significant differences (* *p* < 0.05; ** *p* < 0.01) between the treatments (*n* = 14 per treatment).

**Figure 8:**
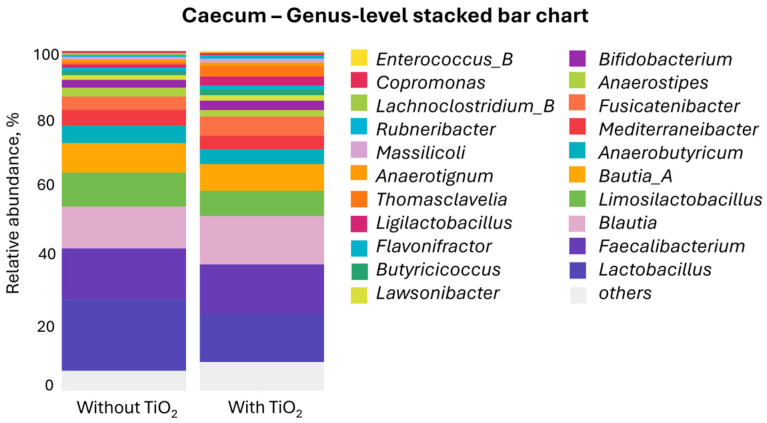
Taxonomic composition of the microbiota at the genus level in caecal digesta samples collected on day 32 from broilers fed diets with or without TiO_2_ supplementation. Relative abundances are expressed as percentages of the total bacterial community. In TiO_2_-supplemented samples, the genera *Thomasclavelia*, *Massilicoli*, *Blautia*, *Rubneribacter*, and *Mediterraneibacter* accounted for 3.2%, 1.1%, 14.2%, 1.1%, and 4.0% of the total bacterial community, respectively.

LEfSe analysis identified five bacterial species that were significantly enriched in caecal digesta samples from birds fed the TiO_2_-supplemented diet: *Thomasclavelia merdavium* with relative abundance reaching 2.0% (*p* = 0.024), *Massilicoli timonensis* (*p* = 0.028, 1.0%), *Blautia merdavium* (*p* = 0.006, 0.75%), *Rubneribacter badeniensis* (*p* = 0.008, 1.0%), and *Mediterraneibacter caccavium* (*p* = 0.003, 0.5%) (Figure 6 and Figure 7). Among these species, *T. merdavium* exhibited the greatest difference in relative abundance, with a median abundance of 2.0% in TiO_2_-supplemented samples compared with 0.5% in samples without TiO_2_ (Figure 7A). At the genus level, *Thomasclavelia* accounted for an average relative abundance of 3.2% in TiO_2_ supplemented samples and 0.9% in control samples (Figure 8). The taxonomic composition of the caecal microbiota at the family level in birds fed diets with or without TiO_2_ supplementation is shown in Supplementary Figure S2.

### 3.3. Fermentation Responses in Ex Vivo Experiment II: Caecal Model

#### 3.3.1. Gas Production

**Figure 9:**
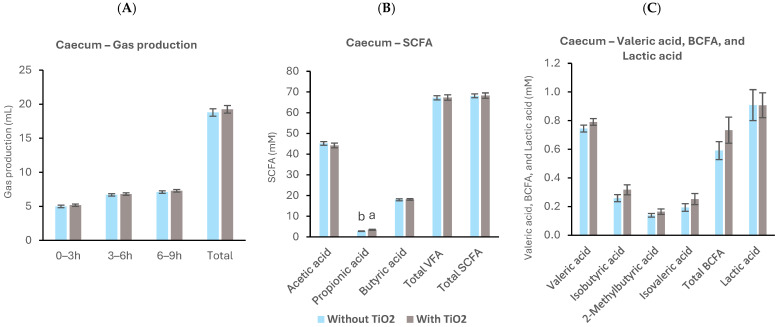
Effects of TiO_2_ supplementation on fermentation activity in the caecal ex vivo model II. (**A**) Gas production (mL). (**B**) Acetic acid (mM), propionic acid (mM), butyric acid (mM), total VFA (mM), and total SCFA (mM) concentrations. (**C**) Valeric acid (mM), isobutyric acid (mM), 2-methylbutyric acid (mM), isovaleric acid (mM), total BCFA (mM), and lactic acid (mM) concentrations measured at the end of the 9-h fermentation period (n = 10 per treatment). For gas production, columns show gas production during individual fermentation intervals and total cumulative gas production. Significant differences (*p* < 0.05) are indicated by different letters above each column. Bars represent standard error of the mean.

To assess the effect of microbiota adapted to TiO_2_ on fermentation, substrates and inocula were collected from birds fed either a control diet or a diet supplemented with TiO_2_. The effects of microbiota adaptation to TiO_2_ on caecal fermentation are shown in Figure 9.

In the second caecal ex vivo simulation, gas production was measured over a 9-h fermentation period (Figure 9A). No statistically significant differences were observed between treatments during the 0–3 h, 3–6 h, or 6–9 h intervals, or for cumulative gas production over the 0–9 h period.

#### 3.3.2. Fermentation End-Product Concentrations

TiO_2_ supplementation resulted in differences in SCFA concentrations compared with the control treatment (Figure 9B). Specifically, propionic acid concentration was higher in TiO_2_ treatment (*p* < 0.05). Moreover, total and individual BCFAs exhibited trends similar to those observed in the ex vivo experiment I, where TiO_2_ was directly added to the fermentation vessels, but no other significant effects were observed (Figure 9C).

## 4. Discussion

This study evaluated the effects of TiO_2_, a commonly used inert dietary marker, on microbial fermentation and microbiota activity in the ileum and caecum of broilers using combined ex vivo and in vivo approaches. In addition, the ex vivo model was refined by incorporating substrates and microbiota from birds previously exposed to TiO_2_ to better reflect in vivo conditions. TiO_2_ altered microbial fermentation in a segment-specific manner, reducing gas production and acetate formation in the ileum while increasing gas production and branched-chain fatty acids in the caecum.

In ex vivo systems, gas production reflects microbial fermentation activity [16,19]. The observed changes following TiO_2_ inclusion indicate segment-specific responses consistent with the distinct ecology of the ileum and caecum. In the ileal ex vivo model, TiO_2_ reduced gas production, indicating that the addition of TiO_2_ directly altered microbial fermentation under otherwise identical conditions. This finding suggests a suppression of microbial fermentation under ileal conditions [20,21]. In contrast, gas production increased in the caecum, the principal site of microbial fermentation in broilers [14,21]. This may reflect the greater diversity and metabolic capacity of caecal microbiota, which may contribute to a different response to TiO_2_ compared to the ileum.

Fermentation end-products further support segment-specific microbial responses to TiO_2_. In the ileal ex vivo model, TiO_2_ treatment resulted in a reduction in acetic acid concentration, consistent with the decrease in gas production and suggesting reduced carbohydrate fermentation. Because the ileal and caecal fermentations were assessed independently, this finding cannot be directly linked to substrate flow into the caecum; however, it indicates that TiO_2_ can modify microbial metabolic activity in the proximal gut. In the caecal ex vivo model, addition of TiO_2_ consistently increased BCFA concentrations, namely isobutyrate, 2-methylbutyrate and isovalerate, compared with the control treatment. When fermentable carbohydrates are limited, protein can become a major substrate for caecal microbiota [20]. Under these conditions, bacterial degradation generates not only BCFAs but also a spectrum of aromatic metabolites, including indole, skatole, phenol and p-cresol, several of which are toxic or carcinogenic to the host [22]. Together, these findings suggest that TiO_2_, despite its widespread use as an inert tracer, may modulate nitrogen flow or microbial metabolic preferences in a site-specific manner.

Total eubacterial counts in the ileal ex vivo model were not affected by TiO_2_, indicating no change in overall bacterial abundance. However, as only total counts were measured, shifts in community composition cannot be excluded, and effects are likely selective, consistent with previous reports of taxon-specific responses to TiO_2_ [23]. In the caecal ex vivo model, TiO_2_ increased total eubacterial concentrations, suggesting differences on microbial proliferation or activity, potentially reflecting changes on the substrate utilisation or microbial function.

In the in vivo setting, TiO_2_ did not affect growth performance or organ development, indicating no overt physiological effects under the conditions tested. However, consistent with ex vivo findings, BCFA concentrations were increased in caecal digesta, supporting a shift towards proteolytic fermentation. This may reflect altered substrate availability or microbial activity in the presence of TiO_2_ [24,25,26,27].

Several taxa were enriched in the caecal digesta of TiO_2_-treated birds at day 32, including *Thomasclavelia merdavium*, *Massilicoli timonensis*, *Blautia merdavium*, and *Rubneribacter badeniensis*. These taxa belong to phylogenetically diverse groups with potential roles in gut metabolism, although their specific functional contributions remain incompletely characterised. While some members of these groups have been associated with amino acid and fermentation pathways, direct involvement in BCFA production at the species level remains to be experimentally validated [28,29,30,31]. Including metagenome-assembled genome (MAG)-derived taxa improves the representation of microbial diversity and supports more robust inference of microbial function, particularly for uncultured taxa [32,33,34,35].

Integrating two ex vivo approaches with in vivo data improved the interpretation of microbial responses to TiO_2_ by distinguishing between acute and adaptation-dependent effects. In the ex vivo experiment I, TiO_2_ was added directly to fermentation vessels, representing an acute exposure scenario. In contrast, the adapted ex vivo experiment II utilised inoculum and substrate from birds previously exposed to TiO_2_, thereby reflecting a microbiota that had undergone in vivo adaptation. Although the differences observed in the adapted model were not statistically significant, the direction of change was consistent with both the standard ex vivo model and in vivo observations, particularly with respect to BCFA production. This consistency suggests that TiO_2_ may exert reproducible effects on microbial metabolic activity, while also highlighting the importance of microbial adaptation in modulating the magnitude of these responses. As microbial communities dynamically adjust to dietary inputs [15,23,36], combining in vivo and ex vivo approaches provides a more physiologically relevant framework for assessing the effects of dietary markers. Collectively, these findings suggest that TiO_2_ may influence microbial fermentation dynamics within the gastrointestinal tract and may therefore not be entirely biologically inert, warranting further investigation in animal studies.

## 5. Conclusions

Ex vivo models provide a physiologically relevant platform to study gut microbiota in their native ecological context, including uncultured populations. Using microbial inocula from birds adapted to dietary interventions improves ex vivo model understanding by reflecting microbiota that have already undergone in vivo adaptation. In this study, combined ex vivo and in vivo approaches produced consistent results, indicating that TiO_2_ moderately influences microbial fermentative activity irrespective of exposure method. These findings highlight the importance of microbial adaptation and dietary context in interpreting ex vivo data and support their integration with in vivo models to better evaluate gut microbial function and nutritional strategies.

## Figures and Tables

**Table 1:** Growth performance of broilers fed without and with TiO_2_ diets from 0–14, 14–21, and 0–21 days of age ^1^.

	Day 0–14				Day 14–21				Day 0–21		
	FI (kg/pen)	BWG (g)	mFCR	BW (g)	FI (kg/pen)	BWG (g)	mFCR	BW (g)	FI (kg/pen)	BWG (g)	mFCR
Without TiO_2_	7.85	464.00	1.07	509.35	8.56	436.78	1.15	945.78	16.42	899.42	1.15
With TiO_2_	8.08	462.35	1.09	507.42	8.76	449.071	1.25	957.42	16.85	911.14	1.17
SEM	0.130	7.115	0.012	7.233	0.127	10.898	0.022	16.709	0.226	16.667	0.014
*p*-value	0.225	0.855	0.061	0.852	0.275	0.377	0.893	0.622	0.193	0.615	0.234

^1^ FI: feed intake; BWG: body weight gain; mFCR: mortality corrected feed conversion ratio; BW: body weight. Data are means of 14 replicate pens per treatment.

**Table 2:** Growth performance of broilers fed without and with TiO_2_ diets from 21–32, and 0–32 days of age ^1^.

	Day 21–32				Day 0–32		
	FI (kg/pen)	BWG (g)	mFCR	BW (g)	FI (kg/pen)	BWG (g)	mFCR
Without TiO_2_	20.48	844.78	1.54	1790.00	36.91	1744.78	1.34
With TiO_2_	20.56	814.57	1.63	1771.57	37.40	1726.21	1.38
SEM	0.215	10.845	0.023	25.20	0.408	25 3	0.015
*p*-value	0.813	0.060	0.099	0.610	0.402	0.607	0.31

^1^ FI: feed intake; BWG: body weight gain; mFCR: mortality corrected feed conversion ratio; BW: body weight. Data are means of 14 replicate pens per treatment.

**Table 3:** Effect of TiO_2_ inclusion on absolute and relative weight ^1^ of internal organs and different parts of GIT of broilers at 14 d, 21 d and 32 d of age.

	Day 14												
	BW ^2^	Crop	R-Crop	Gizzard	R-Gizzard	Liver	R-Liver	Duodenum	R-Duodenum	Jejunum	R-Jejunum	Ileum	R-Ileum
Without TiO_2_	527.25	2.71	0.51	12.42	2.42	18.73	3.57	21.42	4.19	52.21	10.23	53.10	10.41
With TiO_2_	522.00	2.69	0.52	12.44	2.39	19.18	3.58	21.50	4.15	51.16	9.91	52.26	10.07
SEM	15.482	0.111	0.016	0.346	0.071	0.855	0.093	0.327	0.125	1.124	0.335	0.996	0.323
*p*-value	0.814	0.918	0.762	0.963	0.802	0.704	0.936	0.876	0.812	0.513	0.512	0.558	0.457
	**Day 21**												
Without TiO_2_	895.42	3.59	0.39	18.11	1.97	26.85	2.99	22.60	2.49	58.92	6.67	55.10	6.20
With TiO_2_	918.64	3.72	0.40	17.91	2.04	26.92	2.93	22.85	2.58	59.23	6.51	57.60	6.35
SEM	23.604	0.145	0.016	0.492	0.062	0.925	0.055	0.323	0.055	1.213	0.172	1.473	0.185
*p*-value	0.481	0.427	0.583	0.761	0.401	0.961	0.483	0.592	0.271	0.858	0.501	0.232	0.561
	**Day 32**												
Without TiO_2_	1864	5.07	0.27	24.56	1.34	48.23	2.60	26.00	1.38	67.42	3.64	63.69	3.22
With TiO_2_	1866	5.65	0.30	25.01	1.35	48.49	2.58	25.67	1.40	67.53	3.62	59.96	3.43
SEM	34.766	0.280	0.014	0.840	0.063	1.239	0.044	0.485	0.032	1.283	0.065	1.337	0.074
*p*-value	0.963	0.151	0.113	0.707	0.911	0.880	0.818	0.641	0.625	0.953	0.902	0.051	0.051

^1^ R: relative weight, was calculated by dividing absolute weight by body weight of each sampled bird. Data are means of 2 birds per pen and 14 replicate pens per treatment. ^2^ BW: Mean body weight of the two birds that were sacrificed for internal organ measurements.

## Data Availability

The data presented in this study is available on request from the corresponding author. The raw shotgun metagenomic sequencing data generated in this study have been deposited in the National Center for Biotechnology Information (NCBI) Sequence Read Archive (SRA) under BioProject accession number PRJNA1477333. The dataset comprises 48 metagenomic samples, available under SRA run accession numbers SAMN60753512–SAMN60753559. The data will be publicly accessible upon publication of this manuscript. Additional processed data is available from the corresponding author upon a reasonable request.

## Supplementary Materials

The following supporting information can be downloaded at: https://www.mdpi.com/article/10.3390/ani16121867/s1, Table S1: Ingredient composition and nutrient content of the diets from 0–21 d and 21–32 d of age (%, as-fed basis); Figure S1: (A) Comparison of the alpha diversity (Chao1 index) of caecal microbiota among treatments with and without TiO_2_. (B) Comparison of the alpha diversity (Simpson index) of caecal microbiota among treatments with and without TiO_2_; Figure S2: The bar plot presented delineates the taxonomic distribution of bacteria in the caecum on the Family level across samples with and without TiO_2_ addition.
