# Supplementary material for: Investigation of the Effect of TiO2 as a Dietary Marker on Broiler Intestinal Fermentation: Combination of Ex Vivo Simulation and In Vivo Approach
Source: Animals (Basel). 2026 Jun 17;16(12):1867. doi: 10.3390/ani16121867 (PMC13296173; doi:10.3390/ani16121867)
Supplement: Supplementary file 1 [file animals-16-01867-s001.zip › animals-4355170-supplementary.pdf]

Supplementary Table S1. Ingredient composition and nutrient content of the diets from 0-21d and 21-32 d of age (%, as-fed basis).

**Ingredient** (%)	**Starter-Grower (0-21d)**	**Finisher (21-end d)**
Wheat	65.97	75.01
Soybean meal 48	27.27	17.78
Soy oil	3.65	4.28
Salt	0.35	0.35
DL Methionine	0.24	0.27
Lysine HCl	0.23	0.30
Threonine	0.09	0.15
Limestone	1.13	1.19
Mono Ca Phosphorus	0.66	0.26
Phytase[1]	0.01	0.01
Vit+Mineral premix[2]	0.40	0.40
TiO_2	Without/With 0.04	Without/With 0.04
**Calculated chemical composition** (%)		
Crude protein	21.75	18.96
ME kcal/kg	3025.00	3150.00
Calcium	0.90	0.83
Phos	0.71	0.62
Avail Phos	0.49	0.39
Digestible Met	0.51	0.48
Digestible Lys	1.19	1.08
Digestible Tryp	0.21	0.19
Digestible Thr	0.82	0.73

[1] Quantum® Blue (AB Vista, Marlborough, Wiltshire, UK) supplied at 500FTU phytase/kg of diet. [2] Vitamin and mineral premix provided (per kilogram of diet): vitamin A 13,000 IU; vitamin D3 5,000 IU; vitamin E 80,000 IU; vitamin K3 3.2 mg; vitamin B1 3.2 mg; vitamin B2 8.6 mg; vitamin B6 5.4 mg; vitamin B12 0.017 mg; biotin 0.3 mg; folic acid 2.2 mg; niacinamide 60 mg; D-pantothenic acid 17 mg; Fe (as FeSO4) 20 mg; Cu (as CuSO4) 15 mg; Mn (as MnO) 120 mg; Zn (as ZnO) 54 mg; I (as Ca(IO3)2) 1.25 mg; Se (as Na2SeO3) 0.297 mg; calcium as calcium carbonate (carrier) 26.4 mg.

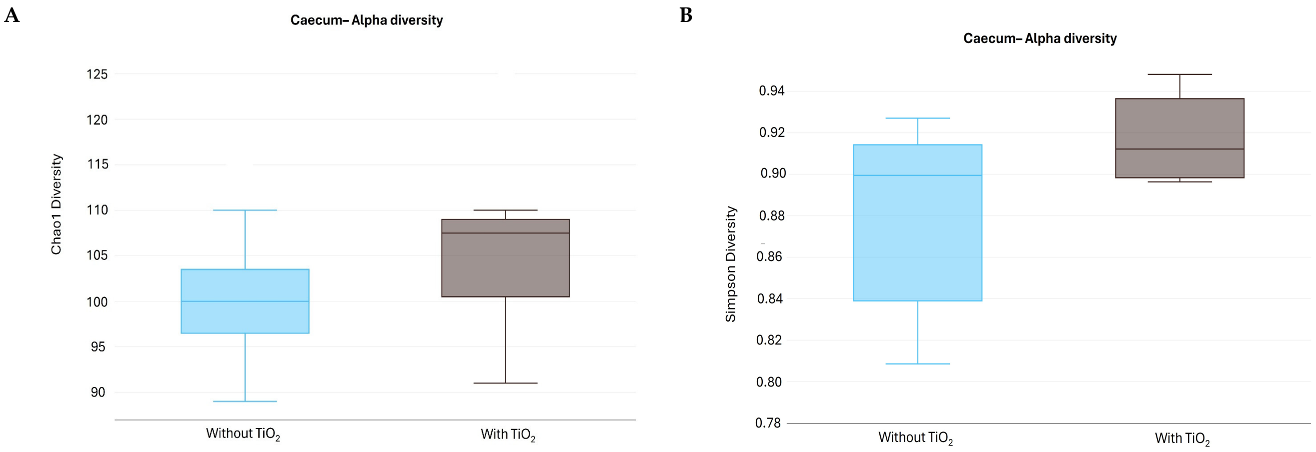

Supplementary Figure S1. (A) Comparison of the alpha diversity (Chao1 index) of caecal microbiota among treatments with and without TiO_2. (B) Comparison of the alpha diversity (Simpson index) of caecal microbiota among treatments with and without TiO_2.

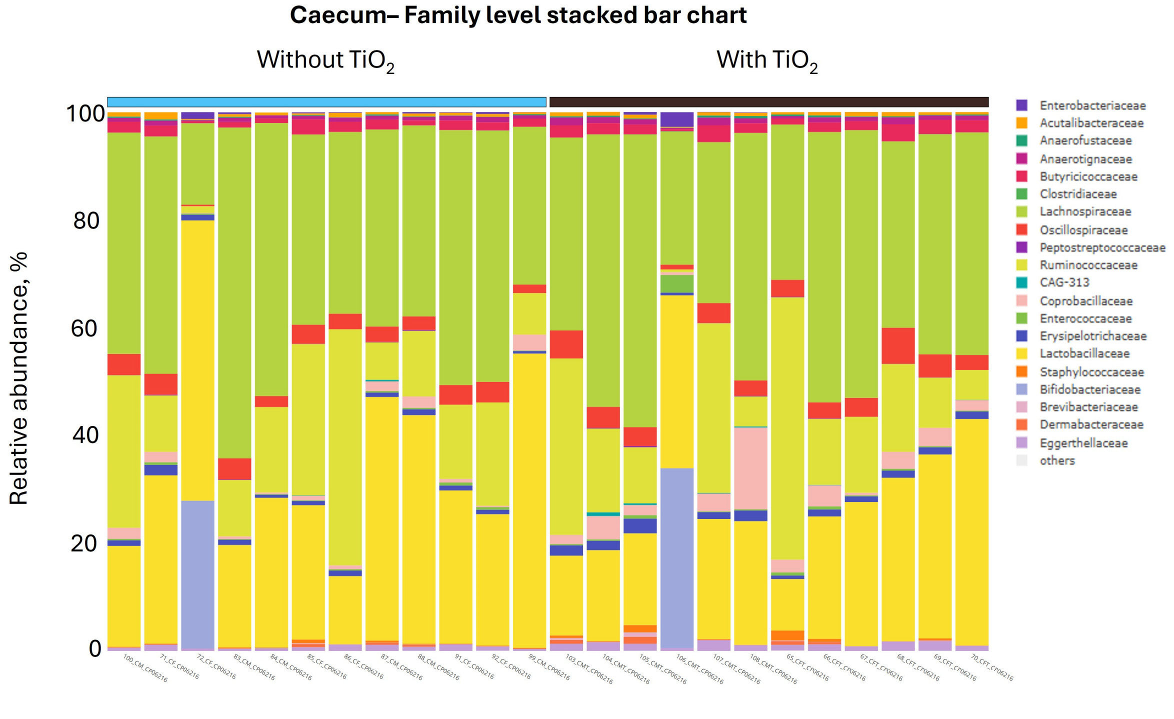

Supplementary Figure S2. The bar plot presented delineates the taxonomic distribution of bacteria in the caecum on the Family level across samples with and without TiO2 addition.
